# Trimethoprim-Sulfamethoxazole-Induced Aseptic Meningitis: A Rare Presentation of Commonly Used Antibiotic

**DOI:** 10.1155/2019/4289502

**Published:** 2019-12-11

**Authors:** Salem Agabawi

**Affiliations:** ^1^Department of Internal Medicine, Section of Infectious Diseases, Max Rady College of Medicine, University of Manitoba, Winnipeg, Manitoba, Canada; ^2^Department of Internal Medicine, Section of Infectious Diseases, King AbdulAziz University, Jeddah, Saudi Arabia

## Abstract

Drug-induced aseptic meningitis is a rare medical condition with trimethoprim-sulfamethoxazole being one of the most common antimicrobial agents associated with it. Here, I report a case of a 56-year-old male who presented to a health care facility with shock and meningitis-like syndrome in two occasions, one year apart following an exposure to trimethoprim-sulfamethoxazole for treatment of skin/soft tissue infection. Investigations did not reveal an infectious etiology in the two presentations. The patient improved with supportive care and withdrawal of the offending agent. In the two admissions, the patient improved following stopping the offending drug in addition to supportive care. The diagnosis of trimethoprim-sulfamethoxazole-induced aseptic meningitis was the most likely explanation for this case. Trimethoprim-sulfamethoxazole-induced aseptic meningitis is rare although it is a life-threatening side effect of TMP/SMX; therefore, the clinicians should keep the diagnosis of drug-induced aseptic meningitis in the differential diagnosis of aseptic meningitis in the appropriate clinical setting as early withdrawal of the culprit drug and supportive measurements will lead to early recovery.

## 1. Introduction

Meningitis is an inflammatory condition of the leptomeninges that is usually caused by a bacterial or viral or less likely fungal infection. Aseptic meningitis is a medical term used for patients who have clinical/laboratory evidence of meningeal inflammation in the absence of positive routine bacterial cultures. There are many etiologies for aseptic meningitis, viral-related aseptic meningitis, and in particular, enteroviruses group being the most common identified viruses [1]. Other infectious etiologies include mycobacterial, fungi, spirochetes, and parasites, and least possible etiologies are related to medications, malignancy, autoimmune disorder, and vaccines [2]. Drug-induced aseptic meningitis (DIAM) has been reported in the literature from different classes of medications [3–8]. Here, I report a case of recurrent DIAM related to TMP/SMX in a patient with no previous history of sulfamethoxazole allergy.

## 2. Case Presentation

A 56-year-old male presented to a tertiary care hospital emergency room (ER) in Winnipeg, Canada, with a one-day history of bilateral eye swelling and erythema, whole body swelling, generalized body itching, crampy abdominal pain, nausea, and vomiting. Few days before the presentation, the patient developed folliculitis over the buttock. Superficial skin swab results yielded *coagulase-negative staphylococci (CoNS)* that was susceptible to trimethoprim/sulfamethoxazole (TMP/SMX). The patient was treated with TMP/SMX for presumed skin/soft tissue infection (SSTI) on the day of presentation, and within 60 minutes of taking the medication, he developed the abovementioned symptoms. The patient presented to the health care facility with initial vitals showing blood pressure (BP) of 132/67 mmHg, heart rate (HR) of 110 bpm, respiratory rate of 28 per minute, and temperature of 36.7°C. At presentation, the patient was agitated, restless, and had rigors and neck stiffness; therefore, he was started on Ativan and haloperidol. Two hours later, BP dropped to 80/50 mmHg and temperature raised to 40°C. Blood cultures were withdrawn and started on ceftriaxone, vancomycin, and acyclovir for presumed meningoencephalitis. Due to undifferentiated shock at presentation, 4 litres of crystalloid fluid was administered along with intravenous (IV) steroids and norepinephrine for presumed septic shock. The patient was transferred to Saint-Boniface general hospital (SBGH) for further care. Upon arrival to the ER, vital signs showed BP of 131/82 mmHg on norepinephrine 0.2 mcg/kg/min, HR of 65 bpm, RR of 16/minute, and oxygen saturation 97% on room air (RA). An urgent computed tomography (CT) scan of the brain was performed and revealed no abnormality, followed by urgent lumbar puncture (LP). Cerebrospinal fluid (CSF) revealed a total nucleated cell count (TNCC) of 670 × 10^6^/L with 88% neutrophils and 12% monocytes (normal: 0 to 5 × 10^6^/L), a total protein of 0.98 g/L (normal: 0.2 to 0.4 g/L), and a glucose of 3.9 mmol/L (normal: 2.3 to 4.7 mmol/L). Other pertinent laboratory investigations revealed a peripheral leukocytosis of 18.4 × 10^9^/L (normal: 4.5 to 11.0 × 10^9^/L) with a neutrophil predominance 87% of total leukocyte counts. Blood culture was withdrawn and yielded negative results. The CSF Gram stain was subsequently reported as 4 + PMN, negative culture for bacteria. Of note, *acid-fast Bacilli (AFB)* stain/culture, fungal culture, and *herpes simplex virus (HSV)* polymerase chain reaction (PCR) on CSF were not performed at this time. The patient was continued on ceftriaxone, vancomycin, and acyclovir (at meningitis dose) for presumed meningoencephalitis and admitted to the medical ward. On day two after admission, the infectious diseases (ID) team was consulted for further management of meningoencephalitis. Further chart review, history, and physical exam of the patient revealed that he had an admission 1 year prior with meningitis and shock within two weeks after taking two days of TMP/SMX for presumed *methicillin-sensitive Staphylococcus aureus- (MSSA-)* related SSTI. At the time, he presented with abdominal pain, nausea, vomiting, and fever. He was treated at ER with IV fluid and discharged home. The patient presented one week later with headache, nausea, vomiting, and low BP. He had an LP with CSF analysis revealed a TNCC of 196 × 10^6^/L with 83% neutrophil predominance, 15% monocytes (normal: 0 to 5 × 10^6^/L), a total protein of 1.11 g/L (normal: 0.2 to 0.4 g/L), and a glucose of 4.7 mmol/L (normal: 2.3 to 4.7 mmol/L). The CSF bacterial, fungal, and AFB cultures yielded negative results. Also, CSF HSV and *Cytomegalovirus* (CMV) PCR were negative along with negative *West Nile virus (WNV)* Ab. The patient was treated with seven days of ampicillin, ceftriaxone, and vancomycin followed by an additional ten days of amoxicillin/clavulanic acid as an outpatient. Other relevant medical history was brucellosis infection in 1990 related to walrus meat consumption; however, further details of the diagnosis were not available. The patient denied any previous history of drug allergy. Clinical examination revealed normal vital signs, no evidence of meningeal irritation, and no further generalized body swelling. The ID team concluded that a recurrent TSIAM is a diagnosis in this case. Therefore, further antimicrobials were not warranted and hence discontinued. An allergy/immunology team was consulted for a confirmation of the diagnosis. The allergy team concluded that due to absence of appropriate skin patch testing to prove or disprove the TSIAM at our hospital, a presumed diagnosis of recurrent TSIAM was established based on the current presentation. The patient was advised to avoid TMP/SMX and any sulfa component-containing medications. The team was advised to provide a medical alert bracelet to the patient to avoid TMP/SMX in the future. The patient was discharged home on day 5 after admission.

## 3. Discussion

DIAM is a rare clinical entity with diagnosis being established based on the exclusion of other etiologies and the causal relationship with a culprit drug. The most commonly identified medication in DIAM is the nonsteroidal anti-inflammatory drugs (NSAIDs) with ibuprofen being the most likely observed NSAIDs among others. Other medications were observed including but not limited to intravenous immunoglobulin (IVIG), anticonvulsants, monoclonal antibodies, allopurinol, azathioprine, vaccines, and antimicrobial drugs [6–8].

Among antimicrobial drugs, TMP/SMX was noted to be one of the most commonly observed drug in the previous reports. Other antimicrobials that have been implicated include B-lactam penicillin, cephalosporin, rifampicin, vancomycin, and metronidazole [7, 8]. A recent review by Bruner et al. [9] revealed 41 cases of TSIAM in the literature. The mechanism of the reaction was not fully understood; however, a possible mechanism was thought to be related to type II hypersensitivity reaction with immune complex deposition as this was noted in the serum of three patients in addition to the faster subsequent reaction following a reexposure TMP/SMX [10, 11]. Another plausible mechanism is related to type IV hypersensitivity with a concept of pharmacologic interaction of the drugs with immune receptors. The concept states that the drug can reversibly bind to the human leukocyte antigen (HLA) on the T-cell receptor and stimulates a variety of type IV T-cell responses [12].

The most common reported clinical findings of TSIAM were headache, fever, neck pain, and altered mental status. The severe reactions, although rare, were hypotension, seizure, decrease level of consciousness, and coma. The typical CSF findings include neutrophil-predominant pleocytosis, elevated protein, and normal glucose. Symptoms usually resolve within 96 hrs, following the withdrawal of the offending medication. Diagnosis can be confirmed with drug challenge or graded test dosing [9].

In this case report, the patient had no previous history of drug allergy in the first admission and presented to the health care facility subacutely within two weeks of drug exposure; therefore, the diagnosis was difficult to make although the CSF analysis did not reveal a definitive cause of meningitis. The second presentation confirmed the diagnosis as the patient challenged himself with the culprit drug and developed the same reaction within 1 hour, which indicate that he was sensitized from the first episode. Subsequently, he recovered within 48 hrs of supportive care and discontinuation of the culprit drug. Therefore, TSIAM is the most likely diagnosis.

In conclusion, I report a rare although a life-threatening drug reaction in a patient presented with recurrent TSIAM. The clinicians should keep the diagnosis of DIAM in the differential diagnosis of aseptic meningitis when there is a temporal association with a culprit drug.
